# Effects of Participation in a Community‐Based Food Cooperative on Food Security and Dietary Behaviours: A Pre‐Post Study

**DOI:** 10.1002/hpja.70272

**Published:** 2026-10-27

**Authors:** Holly Hliounakis, Katherine Kent, Cristy Brooks, Jordan Scavera, Catharine Fleming, Nicky Morrison, Lei Si, David Simmons, Jason H. Y. Wu, Lucy Chapman, Freya MacMillan

**Affiliations:** ^1^ Division of Research and Innovation Western Sydney University Sydney New South Wales Australia; ^2^ School of Medical, Indigenous and Health Sciences, Faculty of Science, Medicine and Health, University of Wollongong Wollongong New South Wales Australia; ^3^ School of Health Sciences, Faculty of Health, Western Sydney University Sydney New South Wales Australia; ^4^ Translational Health Research Institute, Western Sydney University Sydney New South Wales Australia; ^5^ Diabetes Obesity Metabolism Translational Research Unit Western Sydney University Sydney New South Wales Australia; ^6^ School of Medicine, Faculty of Health, Western Sydney University Sydney New South Wales Australia; ^7^ Urban Transformations Research Centre, Western Sydney University Sydney New South Wales Australia; ^8^ The George Institute for Global Health, Faculty of Medicine and Health, University of New South Wales Sydney New South Wales Australia; ^9^ School of Population Health, Faculty of Medicine and Health, University of New South Wales Sydney New South Wales Australia

**Keywords:** alternative food network, Australia, community food cooperative, dietary intake, food insecurity, meal preparation

## Abstract

**Introduction:**

Community‐based food cooperatives have shown potential in addressing food insecurity, but longitudinal evidence assessing their impact is limited. This study evaluated the effects of participating in a community‐based food cooperative on food insecurity, dietary behaviours, and physical health outcomes among Australian adults.

**Methods:**

A pre‐post survey of new cooperative members measured demographics, household food insecurity status (United States Department of Agriculture 6‐item Household Food Security Survey Module (HFSSM)), dietary behaviours (servings/day of core food groups), and objective physical health indicators (weight, waist circumference, and blood pressure). Participants were classified as secure (HFSSM score = 0) or insecure (HFSSM score ≥ 1). A McNemar's test assessed the change in proportion of participants classified as food insecure from baseline to follow‐up, and paired *t*‐tests or non‐parametric equivalents compared other outcomes.

**Results:**

At baseline, 58 participants completed surveys, and 25 completed physical data collection. Participants were primarily female (93.1%), 18–33 or 35–44 years old (37.9% each), and of European background (81.0%). The proportion of participants reporting food security improved from baseline (54.2%) to follow‐up (66.7%) (*p* = 0.041). Importantly, participants who were food secure remained so at follow‐up. Those who were food insecure also reported more favourable improvements in dietary behaviours and meal preparation habits, though many of these did not reach statistical significance.

**Conclusion:**

Participation in a community‐based food cooperative was associated with improved food security among Australian adults.

**So What?:**

These findings highlight the potential of community‐based food cooperatives to reduce food insecurity and support their integration into broader health promotion and food security strategies.

## 1 Introduction

Food insecurity is a recognised public health challenge in high‐income countries, including Australia, and affects 1 in 8 households [1]. It is defined as inadequate access to enough food for an active, healthy life, and exists when access to nutritionally adequate food is limited, or unable to be acquired in safe and socially acceptable ways [2]. Alongside simply having enough food, food security encompasses food accessibility, utilisation, stability, agency, and sustainability, and therefore also requires the ability, resources, and empowerment to make meaningful choices about what food is eaten, how it is sourced, and under what conditions [3].

Food insecurity has well‐documented detrimental implications for health and wellbeing. Recent Australian studies have found that higher levels of household food insecurity are associated with poorer diet quality, physical and mental health [4, 5, 6]. Additionally, food insecurity has been linked to social isolation and loneliness [7]. Addressing food insecurity therefore requires approaches that go beyond food provision to support nutritional adequacy, social connection, and participation [8].

In response to this challenge, community‐based food initiatives are being explored as sustainable options for improved food access, community empowerment, and enhanced wellbeing [9]. Community food cooperatives are member‐based organisations where individuals purchase food collectively, often sourced directly from producers or wholesalers [10]. These models aim to increase agency and shared decision‐making, and foster social connection, enabling people to take an active role in their own food systems [11, 12]. They can also generate health and behaviour change benefits, often outperform supermarkets on resource‐use efficiency and emissions, and are economically viable [13]. These systems have also reported improvements in food security and wellbeing among food‐insecure, low‐income households [14], and have noted reconnection between consumers and producers through community collaboration and collective problem solving [15, 16].

Box Divvy is an Australian organisation that facilitates the establishment of community‐based food cooperatives throughout New South Wales (NSW), the Australian Capital Territory, Victoria, and Queensland. Box Divvy operates through household‐based cooperatives, connecting them with local farmers and wholesalers through an online platform. Members collectively purchase fresh produce and pantry staples in bulk, which Box Divvy estimates saves members up to 30% on fruit and vegetable costs in comparison to conventional supermarkets, supporting food affordability and accessibility by reducing food costs through cutting out intermediaries [17]. A recent retrospective survey of more than 2200 Box Divvy members highlighted substantially lower food insecurity and higher fruit and vegetable intake while using the cooperative compared to before joining, with the largest benefits observed among households experiencing moderate or severe food insecurity [18], highlighting the potential of community‐based food cooperatives as alternative food sources.

To date, research has largely documented the prevalence of food insecurity, but evidence for solutions and interventions targeting food insecurity is scarce [19]. Furthermore, study designs have primarily been cross‐sectional with retrospective data, limiting our understanding of longitudinal or temporal effects of food cooperatives on food insecurity. Finally, there is limited research exploring the implications of food insecurity on other outcomes such as diet quality, meal preparation habits, and objectively measured physical health indicators. To address these gaps, this study aimed to evaluate the effects of Box Divvy participation on food insecurity, dietary behaviours, meal preparation habits, and physical health outcomes among adults in NSW, Australia, using a pre/post study design.

## 2 Methods

### 2.1 Study Design

This study employed a prospective, pre/post design. Participants classified as ‘new’ users residing in the Greater Western Sydney area (Western Sydney, South Western Sydney, Nepean Blue Mountains) were eligible for inclusion. New users were defined as individuals who had signed up to Box Divvy and had either not yet made their first purchase or had made their first purchase within the prior 2 weeks. Baseline survey measurements were collected on a rolling basis between May 2023 and March 2024, with follow‐up measures planned for 6 months after participants commenced purchasing from Box Divvy and taking place between December 2023 and October 2024. Survey participants could opt into physical health measurements, which were also collected on a rolling basis between July 2023 and March 2024, with follow‐up measurements planned after post‐survey completion for each participant, and taking place between January 2024 and July 2025. Informed consent was obtained prior to participants answering the survey questions. Throughout and beyond the study period, participants retained access to Box Divvy. This study was approved by the Western Sydney University Human Research Ethics Committee (approval number H13318).

### 2.2 Measures

#### 2.2.1 Demographic Characteristics

Participant characteristics including gender, age range, ethnic background, household composition, employment status, weekly household estimated income, existing health conditions, and smoking status were self‐reported from multiple choice options. Participants could select multiple options for ethnic background, employment status, and health conditions.

#### 2.2.2 Motivations for Using Box Divvy Reasons and Discontinuation

At baseline, participants were asked ‘What made you want to join Box Divvy?’, and at follow‐up, those who indicated that they were still using Box Divvy were asked ‘What motivated you to continue using Box Divvy over the past 6 months?’ The same multiple‐choice responses were given for both time points and participants were able to select all reasons which applied to them. Participants who indicated that they were no longer using Box Divvy at follow‐up were asked ‘Why did you decide to stop using Box Divvy?’, with the ability to select all multiple‐choice responses which applied to them.

#### 2.2.3 Household Food Insecurity Status

Household food insecurity was measured using the United States Department of Agriculture 6‐item Household Food Security Survey Module (HFSSM). This validated tool includes six questions that measure whether participants have (1) or have not (0) experienced food access challenges over the past 30 days [20]. HFSSM‐6 scores were dichotomised as food secure (score = 0) or experiencing any food insecurity (score = 1–6). This study‐defined classification was selected to distinguish participants reporting no indicators of food insecurity from those reporting at least one affirmative food insecurity response, including more marginal food insecurity. This approach was consistent with our previous research examining food insecurity among members of the same community‐based food cooperative [18].

#### 2.2.4 Dietary Behaviours

Participants self‐reported consumption of fruit, vegetables, protein, grains, dairy, discretionary foods, and water. As per the Australian Short Dietary Screener [21], participants were asked how many serves of each food group they consumed on an average day. Serving sizes were defined and visualised within each survey item. For example, one serve of fruit was described as ‘1 medium apple, 2 small apricots, or 1 cup of chopped melon.’ Response options ranged from ‘never’ to ‘5 or more serves per day.’ Responses were scored from 0 to 5, with ‘never’ coded as 0, ‘less than 1 per day’ coded as 0.5, and values of 1–5 coded according to their numeric value. Alcohol intake was also self‐reported as standard drinks per week, which was divided by seven to reflect daily averages, consistent with the reporting of the other dietary behaviours included in this study.

#### 2.2.5 Meal Preparation Habits

Participants responded to three self‐report questions regarding their food preparation and shopping behaviours over the previous week. They reported the number of meals they had prepared from scratch (e.g., using fresh or minimally processed foods), the number of meals they had not prepared from scratch (e.g., ready meals from the supermarket, or takeaway meals), and the number of supermarket trips they had made during the previous week. Each response was entered as a numerical value into free‐text response boxes.

#### 2.2.6 Physical Health Indicators

Standardised physical health measurements were collected by trained members of the research team at the participant's home or a convenient public space determined by the participant and member of the research team. Systolic and diastolic blood pressure were measured using an iHealth Feel BP5 Wireless Blood Pressure Monitor. Two measurements were taken for all participants, with a third measurement included where there was a > 10% difference between the initial measurements. Participants were seated for five minutes prior to measurement and consecutive measurements were taken two to three minutes apart. Height was measured using a Seca 213 stadiometer, and body weight was measured using Seca 803 digital scales. Waist circumference was measured twice at the mid‐point of the waist, above the hip area, using an anthropometric measuring tape. If measurements differed by > 0.1 cm, a third measurement was taken. Height, weight, and waist circumference were recorded to one decimal point. Where multiple measures were taken, averages were calculated and used in the analysis. Body fat percentage was measured using a Tanita BC‐402 bio‐electrical impedance monitor. Body mass index (BMI) was calculated as (kg/m^2^) using the recorded measurements of height and weight.

### 2.3 Statistical Analysis

Descriptive statistics were calculated for each demographic variable at baseline, as count (*n*) and % of the total sample responding to each multiple‐choice option. These were also calculated to report barriers to having enough food or enough nutritious food, Box Divvy use at follow‐up, intention to continue using Box Divvy, reasons participants ceased Box Divvy use, and reasons participants joined (baseline) and continued to use (follow‐up) Box Divvy. For these data, the differences between groups were assessed through Fishers' exact tests. Additionally, McNemar's tests were conducted to compare participant responses from baseline to follow‐up for reasons to join and continue using Box Divvy. A McNemar's test was also conducted to compare the proportion of participants who were food insecure and secure at baseline and follow‐up time points. Mean (SD) for dietary behaviours, meal preparation habits, and physical health outcomes for baseline, follow‐up and change between baseline to follow‐up are reported for the total sample and by baseline food insecurity status. Paired *t*‐tests for normally distributed data, and Wilcoxon signed‐rank tests for non‐normally distributed data were used to assess change in these outcomes over time. Analyses were conducted using available paired data from baseline and follow‐up. Statistical significance was set at *p* < 0.05. All analyses were conducted in R version 4.4.3 [22].

## 3 Results

### 3.1 Sample Description and Baseline Characteristics

Fifty‐eight new Box Divvy users completed the baseline survey, with 49 (84.5%) completing at least one component of the follow‐up assessment after an average of 6.28 (SD = 0.36) months. Of these participants, 25 also completed physical data collection at baseline, with 17 (68.0%) completing valid follow‐up measurements for at least one physical outcome, after an average of 9.64 (SD = 4.56) months. One participant was excluded from the physical outcome analyses due to recent weight loss surgery. The majority of participants were between 18–34 and 35–44 years of age (each making up 37.9% of the total sample) and of European ethnic background (81.0%). Households primarily comprised of couples with at least one dependent child (58.6%), and participants primarily had education at a university level or higher (56.9%) and were employed on a full‐time (37.9%) or part‐time (25.9%) basis (Table 1). Participants initially heard about Box Divvy through friends/colleagues (41.4%), social media (36.2%), news coverage (10.3%), or through other means (12.0%).

**TABLE 1:** Baseline demographics (*n* = 58).

Category	Variable	*n*	%
Gender	Female	54	93.1%
Age range	18–34	22	37.9%
	35–44	22	37.9%
	45–54	9	15.5%
	55+	5	8.6%
Ethnic background^a^	European	47	81.0%
	South Asian	4	6.9%
	Southeast Asian	8	13.8%
	Māori	1	1.7%
	Aboriginal and/or Torres Strait Islander	2	3.4%
	Other	13	22.4%
Household composition	Couple living alone	9	15.5%
	Couple with non‐dependent child(ren)	2	3.4%
	Couple with dependent child(ren)	34	58.6%
	Person living alone	3	5.2%
	Single parent with dependent child(ren)	5	8.6%
	Multiple family household	4	6.9%
	Other	1	1.7%
Highest level of education	School or intermediate certificate	4	6.9%
	Higher school or leaving certificate	6	10.3%
	Certificate or diploma	15	25.9%
	University degree or higher	33	56.9%
Employment status^a^	Full time employee	22	37.9%
	Part time employee	15	25.9%
	Self employed	9	15.5%
	Employed–unpaid worker	3	5.2%
	In training or education	2	3.4%
	Unemployed, seeking work	5	8.6%
	Unemployed, not seeking employment	8	13.8%
Estimated weekly household income	$0–$999	7	12.1%
	$1000–$1499	14	24.1%
	$1500–$1999	6	10.3%
	$2000–$2999	13	22.4%
	$3000 or more	8	13.8%
	Prefer not to say	10	17.2%
Health conditions^a^	Type 1 diabetes	1	1.7%
	Type 2 diabetes	2	3.4%
	Gestational diabetes^b^	1	1.9%
	Hypertension	8	13.8%
	High cholesterol	5	8.6%
Smoking status	Nonsmoker	40	69.0%
	Previous smoker	13	22.4%
	Currently smoke	2	3.4%
	Currently vape	3	5.2%

^a^ More than one selection available.

^b^ Female denominator.

At baseline, 45.8% of participants were classified as food insecure (*n* = 22). There were no significant demographic differences between groups at baseline (Table S1). Higher proportions of the baseline food insecure group indicated that they had faced barriers to having enough food or enough nutritious food than the food secure group. Specifically, participants who were food insecure at baseline were significantly more likely to report not having enough money for food (*p* < 0.001), having too many other things to pay for (*p* < 0.001), having physical or mental health conditions which stop them from being able to cook or eat properly (*p* < 0.05), and not knowing what food to buy (*p* < 0.05) (Table S2).

### 3.2 Box Divvy Use at Follow‐Up

At follow‐up, 81.6% of participants (*n* = 40) were still using Box Divvy. Of these participants, 100% (*n* = 40) intended to continue using it. At baseline, participants cited reasons for joining Box Divvy. At follow‐up, they reported on whether these reasons were motives for continuing to use Box Divvy (Table 2). Notably, there was a significant increase in participants using Box Divvy to benefit the environment (*p* < 0.05) and to eat more healthily (*p* < 0.05).

**TABLE 2:** Reasons for joining and continuing Box Divvy use.

Category	Baseline *n* (%)	Follow‐up *n* (%)	Change *n* (%)
To benefit the environment through reduced packaging and transport of food	18 (45.0%)	38 (95.0%)	20 (50.0%)^a^
To eat more healthily	15 (37.5%)	26 (65.0%)	11 (27.5%)^a^
To support local farmers and food producers	31 (77.5%)	36 (90.0%)	5 (12.5%)
To be part of a community	5 (12.5%)	9 (22.5%)	4 (10.0%)
To lose weight or prevent a health condition	4 (10.0%)	4 (10.0%)	0 (0.0%)
To access a bigger range of healthy foods	9 (24.4%)	9 (24.4%)	0 (0.0%)
To save money on purchasing healthy foods	34 (85.4%)	30 (75.6%)	−4 (10.0%)

*Note:* Total *n* = 40. Data presented as *n* (% ‘Yes’). McNemar's test conducted to compare baseline vs. follow‐up within participants who said ‘Yes’ to using Box Divvy at follow‐up. More than one selection available.

^a^ Significant at *p* < 0.05.

Participants (*n* = 9) who did not continue to use Box Divvy were able to cite multiple reasons for discontinuing their use. Reasons included not saving enough money when using Box Divvy (44.4%), their hub closing down (33.3%), they didn't like the ordering system (11.1%), did not or could not get the items they wanted (11.1%), found the delivery or pick‐up of orders to be inconvenient (11.1%), found that the frequency of orders didn't work for the household, or cited other reasons (travelling too much to use or were not using the items ordered; 22.2%).

### 3.3 Change in Food Insecurity Status

Food security status improved significantly from baseline to follow‐up, with the proportion of participants reporting food security increasing from 54.2% at baseline to 66.7% at follow‐up (McNemar's test *p* = 0.041). Additionally, all participants who were categorised as food secure at baseline remained food secure at follow‐up (Table 3).

**TABLE 3:** Change in food security status from baseline to follow‐up.

		Follow‐up	
		Food insecure	Food secure
Baseline	Food insecure	16 (33.3%)	6 (12.5%)
	Food secure	0 (0%)	26 (54.2%)

*Note:* Total *n* = 48. Data presented as *n* (% ‘Yes’).

### 3.4 Change in Diet

At both timepoints, the baseline food secure group reported higher daily consumption of vegetables, fruit, grains, protein, dairy, and discretionary foods (Table 4). The full sample reported a statistically significant increase of 0.3 (SD = 1.0) servings of water consumption daily (*p* < 0.05). However, this was attributable to the group who were food insecure at baseline, who reported a statistically significant increase of 0.6 (SD = 1.1) servings per day (*p* < 0.05).

**TABLE 4:** Change in food group servings per day.

	Full sample (*n* = 48)			Food secure at baseline (*n* = 26)			Food insecure at baseline (*n* = 22)		
	Baseline	Follow‐up	Change	Baseline	Follow‐up	Change	Baseline	Follow‐up	Change
Vegetables	2.6 (1.3)	2.8 (1.2)	0.1 (1.0)	3.0 (1.2)	3.0 (1.1)	0.0 (1.2)	2.2 (1.2)	2.5 (1.3)	0.3 (0.9)
Fruit	1.5 (0.8)	1.6 (0.9)	0.1 (0.9)	1.6 (0.8)	1.7 (0.7)	0.1 (0.8)	1.3 (0.7)	1.5 (1.0)	0.2 (0.9)
Grains	2.4 (1.3)	2.3 (1.2)	−0.1 (1.2)	2.5 (1.3)	2.5 (1.2)	0.0 (1.2)	2.3 (1.2)	2.0 (1.1)	−0.3 (1.2)
Protein	1.6 (0.9)	1.6 (1.0)	0.0 (0.8)	1.7 (0.9)	1.7 (1.0)	−0.1 (0.7)	1.5 (0.8)	1.6 (1.0)	0.1 (0.8)
Dairy	1.6 (0.9)	1.6 (0.9)	0.0 (0.9)	1.7 (0.9)	1.6 (0.8)	−0.1 (0.8)	1.5 (1.0)	1.5 (1.1)	0.1 (1.0)
Discretionary	1.4 (0.9)	1.2 (0.8)	−0.1 (1.0)	1.4 (1.0)	1.3 (0.8)	−0.1 (1.1)	1.4 (0.8)	1.1 (0.7)	−0.2 (1.0)
Water	3.5 (1.5)	3.8 (1.4)	0.3 (1.0)*	3.7 (1.5)	3.7 (1.5)	0.0 (0.8)	3.3 (1.6)	3.9 (1.3)	0.6 (1.1)*
Alcohol	0.2 (0.4)	0.2 (0.4)	0.0 (0.3)	0.3 (0.4)	0.3 (0.5)	0.0 (0.3)	0.2 (0.3)	0.1 (0.2)	−0.1 (0.2)

*Note:* Data presented as mean (SD). Paired *t*‐tests conducted for normally distributed data. Wilcoxon signed‐rank tests conducted for non‐normally distributed data.

* *p* < 0.05.

### 3.5 Change in Meal Preparation Habits

Meal preparation habits varied between baseline food insecurity groups. The baseline food insecure group significantly reduced their weekly supermarket trips, from 2.50 (SD = 1.37) to 1.82 (SD = 1.10) (*p* < 0.05). There were no statistically significant changes in the average weekly number of main meals prepared from scratch using fresh or minimally processed foods, or in the number of main meals not prepared from scratch in either group.

### 3.6 Change in Physical Outcomes

From baseline to follow‐up there were no statistically significant changes in physical outcomes (Table 5).

**TABLE 5:** Change in physical outcomes.

Outcome	*n*	Baseline	Follow‐up	Change	*p*
Weight (kg)	16	73.28 (24.57)	73.72 (27.08)	0.44 (3.79)	0.651
BMI	16	26.16 (7.12)	26.34 (7.75)	0.17 (1.31)	0.606
WC (cm)	13	86.68 (19.30)	88.48 (17.91)	1.81 (8.41)	0.454
SBP (mmHg)	14	110.43 (11.30)	109.48 (16.00)	−0.95 (11.62)	0.764
DBP (mmHg)	14	73.20 (6.44)	70.61 (10.40)	−2.60 (9.08)	0.304
Heart rate (bpm)	13	74.09 (6.21)	72.13 (10.54)	−1.96 (10.40)	0.509
Body fat (%)	15	32.59 (10.27)	29.57 (8.20)	−3.02 (11.55)	0.328

*Note:* Data presented as mean (SD). Paired *t*‐tests used to test difference between baseline and follow‐up.

Abbreviations: BMI = body mass index; DBP = diastolic blood pressure; SBP = systolic blood pressure; WC = waist circumference.

## 4 Discussion

This study demonstrated that participation in a community‐based food cooperative was associated with a significant reduction in the proportion of participants experiencing any level of food insecurity over time, alongside sustained food security among other participants. Statistically insignificant but modest, favourable changes in dietary intake, meal preparation behaviours, and food procurement patterns were also noted. Importantly, the benefits were evident for participants experiencing food insecurity, suggesting that community‐based food cooperatives may be particularly effective in supporting vulnerable households by addressing cost and access barriers. Alongside this, over 80% of participants remained Box Divvy users at follow up, and all intended to continue using the platform. These findings highlight the potential of food cooperative models in supporting food security, offering a sustainable and dignity‐based alternative to traditional food relief approaches.

Taken together, the stability observed among food secure households and the improvement among food insecure participants suggests that the model may serve as a supportive resource for all participants, regardless of food security status. This improvement complements existing cross‐sectional evidence which identifies food cooperatives as a promising mechanism for reducing food insecurity [18]. This differential effect reinforces the model's relevance for households at greatest risk, underpinning the potential for community‐based food initiatives to be positioned as preventive measures prior to severe food insecurity and concurring with broader national food security and nutrition policy discussions [9].

Reflecting well‐established gradients in diet quality [5, 23], food secure participants reported healthier dietary habits at both time points. Despite this, those in the food insecure group reported larger improvements for most dietary intake habits and meal preparation habits, including fewer weekly supermarket trips, more scratch‐cooked meals, and fewer meals not prepared from scratch at home. The small but consistent shifts provide early behavioural indications of improved food access and agency, suggesting that affordability may reduce barriers to nutritious food. This is consistent with evaluations of similar models which aim to reduce produce costs and improve access to healthy foods and are associated with higher fruit and vegetable intake [18, 24, 25]. Considering that diet quality is closely related to long‐term health outcomes, the benefits of these structural supports could extend towards healthier trajectories across the lifespan [26, 27], supporting the integration of food cooperatives into wider nutrition promotion strategies.

Contrary to previous research linking food insecurity to higher body weight [28, 29], and poorer physical health outcomes [30, 31, 32, 33], we observed no significant changes in physical health. Recruitment and scheduling of face‐to‐face physical measurements were key challenges due to geographically dispersed participants, which led to a small sample size. Therefore, it is likely that our evaluation of any physical changes had limited statistical power. Alternative options for physical data collection, such as remote delivery of physical measurement devices, or providing assistance for participants to self‐report measures should be tested in future studies to recruit and retain larger sample sizes [34]. This would enable a stronger evaluation of whether change in food insecurity can directly impact these health outcomes.

### 4.1 Strengths and Limitations

This study has several strengths. First, the prospective pre‐post design allowed for the assessment of changes in food security, dietary and food preparation behaviours, and physical health, addressing a key limitation of prior research. Second, the use of validated food security and dietary intake tools alongside objective physical measurements strengthens confidence that our findings reflect true differences arising during the study time period. Despite these strengths, this study is limited by the following factors. The pre‐post design lacked a control group and randomisation, so causal relationships regarding the effect of this food cooperative on the measured outcomes cannot be established. Additionally, high attrition and the use of self‐reported dietary data may reflect potential selection bias, so findings from this study should be interpreted with some caution. Finally, challenges faced regarding physical data collection and an unpowered sample size may have limited our evaluation of physical health changes.

Future research should assess the effects of community food cooperatives with a larger sample size powered to detect meaningful changes in health outcomes. This work should also assess whether access to these cooperatives changes food expenditure patterns and whether any resulting savings are redirected to other household financial needs. Longer‐term follow‐up is also needed to determine the sustainability of changes to food insecurity, meal preparation habits, and dietary change. Finally, additional research is needed to investigate the effect of food cooperatives on other outcomes, such as social connectedness and agency, and how these may impact food security.

### 4.2 Conclusions

This research contributes to the growing evidence that community‐based initiatives can serve as a practical and feasible way to reduce food insecurity. The sustainability of these programs, alongside small but consistent improvements in dietary habits, also shows promise for additional benefits. Therefore, community‐based food cooperatives can function as locally driven additions to the food supply chain, serving as health promotion and dietary behaviour change infrastructure and showing their potential for more than just low‐cost food provisioning.

## Author Contributions

**Holly Hliounakis:** data curation, formal analysis, visualisation, writing – original draft, writing – review and editing. **Katherine Kent:** conceptualisation, methodology, formal analysis, writing – original draft, writing – reviewing and editing, supervision. **Cristy Brooks:** investigation, methodology, project administration, resources, supervision, writing – reviewing and editing. **Jordan Scavera:** data curation, investigation, resources, writing – reviewing and editing. **Catharine Fleming:** conceptualisation, methodology – reviewing and editing. **Nicky Morrison:** conceptualisation, interpretation of data, writing – reviewing and editing. **Lei Si:** conceptualisation, interpretation of data, writing – reviewing and editing. **David Simmons:** conceptualisation, methodology, writing – reviewing and editing. **Jason H. Y. Wu:** interpretation of data, writing – reviewing and editing. **Lucy Chapman:** investigation, writing – reviewing and editing. **Freya MacMillan:** conceptualisation, methodology, writing – reviewing and editing, supervision, funding acquisition.

## Funding

This study was funded through the Western Sydney University Research Theme Champion Grant Assistant Scheme.

## Ethics Statement

This study was conducted according to the guidelines of the Declaration of Helsinki and approved by the Western Sydney University Human Research Ethics Committee (approval number H13318).

## Conflicts of Interest

Freya MacMillan and Cristy Brooks are consumers of Box Divvy. They were not involved in the processing or analysis of data. The other authors declare no conflicts of interest.

## Supporting information


**Table S1:** Demographic differences between food insecurity status at baseline.

**Table S2:** Baseline differences between food insecure and secure participants regarding reasons for not enough food or not enough nutritious food.

## Data Availability

Deidentified participant data and a data dictionary may be made available upon written request to the authors. The data will be accessible from the time of publication, and no end date is currently set for its availability. Access will be granted for research purposes only, subject to the approval of a formal request and a signed data use agreement.
