# Supplementary material for: Effects of Participation in a Community‐Based Food Cooperative on Food Security and Dietary Behaviours: A Pre‐Post Study
Source: Health Promot J Austr. 2026 Oct 27;38(1):e70272. doi: 10.1002/hpja.70272 (PMC13714161; doi:10.1002/hpja.70272)
Supplement: Supplementary file 1 [file HPJA-38-0-s001.docx]

**Supplementary Table 1. Demographic differences between food insecurity status at baseline**

| Category | Variable | Food secure  n (%) | Food insecure  n (%) | *p* value |
| --- | --- | --- | --- | --- |
| Gender | Female | 29 (90.6%) | 25 (96.2%) | 0.620 |
| Age range | 18-34 | 12 (37.5%) | 10 (38.5%) | 0.772 |
|  | 35-44 | 11 (34.4%) | 11 (42.3%) |  |
|  | 45-54 | 5 (15.6%) | 4 (15.4%) |  |
|  | 55+ | 4 (12.5%) | 1 (3.8%) |  |
| Ethnic background* | European | 25 (78.1%) | 22 (84.6%) | 0.738 |
|  | South Asian | 4 (12.5%) | 0 (0.0%) | 0.120 |
|  | Southeast Asian | 5 (15.6%) | 3 (11.5%) | 0.720 |
|  | Māori | 0 (0.0%) | 1 (3.8%) | 0.448 |
|  | Aboriginal and/or Torres Strait Islander | 0 (0.0%) | 2 (7.7%) | 0.197 |
|  | Other | 8 (25.0%) | 5 (19.2%) | 0.754 |
| Household composition | Couple living alone | 5 (15.6%) | 4 (15.4%) | 0.739 |
|  | Couple with non-dependent child(ren) | 1 (3.1%) | 1 (3.8%) |  |
|  | Couple with dependent child(ren) | 21 (65.6%) | 13 (50.0%) |  |
|  | Person living alone | 1 (3.1%) | 2 (7.7%) |  |
|  | Single parent with dependent child(ren) | 2 (6.2%) | 3 (11.5%) |  |
|  | Multiple family household | 1 (3.1%) | 3 (11.5%) |  |
|  | Other | 1 (3.1%) | 0 (0.0%) |  |
| Highest level of education | School or intermediate certificate | 2 (6.2%) | 2 (7.7%) | 0.239 |
|  | Higher school or leaving certificate | 4 (12.5%) | 2 (7.7%) |  |
|  | Certificate or diploma | 5 (15.6%) | 10 (38.5%) |  |
|  | University degree or higher | 21 (65.6%) | 12 (46.2%) |  |
| Employment status* | Full time employee | 11 (34.4%) | 11 (42.3%) | 0.594 |
|  | Part time employee | 8 (25.0%) | 7 (26.9%) | 1.000 |
|  | Self employed | 6 (18.8%) | 3 (11.5%) | 0.495 |
|  | Employed – unpaid worker | 1 (3.1%) | 2 (7.7%) | 0.582 |
|  | In training or education | 1 (3.1%) | 1 (3.8%) | 1.000 |
|  | Unemployed, seeking work | 2 (6.2%) | 3 (11.5%) | 0.648 |
|  | Unemployed, not seeking employment | 6 (18.8%) | 2 (7.7%) | 0.276 |
| Estimated weekly household income | $0 - $999 | 2 (6.2%) | 5 (19.2%) | 0.280 |
|  | $1000-$1499 | 8 (25.0%) | 6 (23.1%) |  |
|  | $1500-$1999 | 4 (12.5%) | 2 (7.7%) |  |
|  | $2,000-$2,999 | 5 (15.6%) | 8 (30.8%) |  |
|  | $3,000 or more | 5 (15.6%) | 3 (11.5%) |  |
|  | Prefer not to say | 8 (25.0%) | 2 (7.7%) |  |

**Note.** *More than one selection available. *P*-values from Fisher’s exact test (full r×2 table for single-choice; 2×2 Yes/No for multi-select).
