# Supplementary material for: Effects of Participation in a Community‐Based Food Cooperative on Food Security and Dietary Behaviours: A Pre‐Post Study
Source: Health Promot J Austr. 2026 Oct 27;38(1):e70272. doi: 10.1002/hpja.70272 (PMC13714161; doi:10.1002/hpja.70272)
Supplement: Supplementary file 2 [file HPJA-38-0-s002.docx]

**Supplementary Table 2. Baseline differences between food insecure and secure participants regarding reasons for not enough food or not enough nutritious food**

| Reason | Food insecure at baseline (n=26) | Food secure at baseline  (n=32) | *P*-value |
| --- | --- | --- | --- |
| Not enough money for food | 10 (38.5%) | 1 (3.1%) | 0.001 |
| Too many other things to pay for | 21 (80.8%) | 7 (21.9%) | <0.001 |
| Food in shops/supermarkets costs too much | 25 (96.2%) | 16 (50.0%) | <0.001 |
| Too hard to get to the store, shops, supermarkets, or market | 8 (30.8%) | 5 (15.6%) | 0.213 |
| The store, shops, supermarket, or market is too far away | 4 (15.4%) | 1 (3.1%) | 0.163 |
| The shops don’t sell or stock nutritious food | 4 (15.4%) | 6 (18.8%) | 1.000 |
| Food that is familiar or appropriate for our culture or religion is not available | 4 (15.4%) | 3 (9.4%) | 0.689 |
| Don’t know what food to buy | 11 (42.3%) | 5 (15.6%) | 0.038 |
| Don’t know how to prepare or cook food | 4 (15.4%) | 1 (3.1%) | 0.163 |
| No place to store food safely | 3 (11.5%) | 2 (6.2%) | 0.648 |
| Don’t have the equipment to prepare or cook food | 3 (11.5%) | 0 (0.0%) | 0.084 |
| Kitchen is not sufficient or safe for cooking | 1 (3.8%) | 1 (3.1%) | 1.000 |
| Not enough time to cook or shop | 12 (46.2%) | 15 (46.9%) | 1.000 |
| Physical or mental health condition that stops me being able to cook or eat properly | 10 (38.5%) | 4 (12.5%) | 0.031 |

**Note.** Data presented as n (%). *P*-values from Fisher’s exact test.
